# Promotion of Bronchopulmonary Dysplasia Progression Using Circular RNA circabcc4 via Facilitating PLA2G6 Expression by Sequestering miR-663a

**DOI:** 10.3389/fcell.2020.585541

**Published:** 2020-10-27

**Authors:** Yu-fei Chen, Dan-dan Feng, Sheng-hua Wu, Hong-yan Lu, Asfia Banu Pasha, Dhivya Lakshmi Permall, Jia-he Chen, Zhong-yi Sun, Bing-jie Li, Huan Zhou, Yang Yang, Xiao-jie Zhang, Xiao-qing Chen

**Affiliations:** ^1^Department of Pediatrics, The First Affliated Hospital of Nanjing Medical University, Nanjing, China; ^2^Department of Pediatrics, The Affiliated Hospital of Jiangsu University, Zhenjiang, China; ^3^Department of Neonatology, Nanjing Children’s Hospital of Nanjing Medical University, Nanjing, China

**Keywords:** circABCC4, miR-663a, PLA2G6, bronchopulmonary dysplasia, lung injury

## Abstract

Circular RNA (circRNA) has been increasingly proven as a new type of promising therapeutic RNA molecule in a variety of human diseases. However, the role of circRNA in bronchopulmonary dysplasia (BPD) has not yet been elucidated. Here, a new circRNA circABCC4 was identified from the Agilent circRNA chip as a differentially expressed circRNA in BPD. The relationship between circABCC4 level and BPD clinicopathological characteristics was analyzed. The function of circABCC4 was evaluated by performing CCK-8 and apoptosis analysis *in vitro* and BPD model analysis *in vivo*. RNA immunoprecipitation (RIP), luciferase reporter and rescue experiments were used to elucidate the interaction between circABCC4 and miR-663a. Luciferase reporter assay and rescue experiments were used to elucidate the interaction between PLA2G6 and miR-663a. CircABCC4 and PLA2G6 levels were increased, while miR-663a levels were decreased in the BPD group, compared to the control group. MiR-663a inhibited apoptosis by repressing PLA2G6 expression, while circABCC4 enhanced the apoptosis and inhibited the proliferation of A549 cells by sponging miR-663a and increasing PLA2G6 expression. In conclusion, circABCC4 promotes the evolving of BPD by spongening miR-663a and up-regulating PLA2G6 expression, which makes circABCC4 an ideal molecular target for early diagnosis and intervention of BPD.

## Introduction

Bronchopulmonary dysplasia (BPD) is a chronic lung disease with high incidence and mortality in premature infants who require mechanical ventilation (Hwang and Rehan, 2018; Tracy and Berkelhamer, 2019). Although many advances have been made in the diagnosis and treatment of BPD, the vast majority of BPD patients still suffer from pulmonary damage that may lead to lung dysfunction in their later life (Principi et al., 2018). Always triggered by multiple factors, the pathogenesis of BPD is complex and remains obscure. However, it has been found that inflammatory response and oxidative stress play their own essential roles (Guaman et al., 2015; Shahzad et al., 2016). Therefore, it is urgent to elucidate the molecular mechanism of BPD before finding new molecular targets for early diagnosis and treatment.

CircRNAs are non-coding RNAs bearing a covalently closed continuous loop between 5′- and 3′-ends (Conn et al., 2015). This circular structure endows circRNAs with a high stability in different species. Previous studies have shown that they are abundant in exosomes and plasma (Li et al., 2015). Research finds that various circular RNAs (circRNAs) are involved in various biological processes, such as cell cycle, proliferation, and apoptosis in diseases (Wang et al., 2019; Yang et al., 2019). At present, there are scarce reports of circRNAs in the animal model of BPD, and previous findings are mainly made through sequencing analyses. The molecular mechanism of circRNAs in BPD needs in-depth investigation (Shen et al., 2019). At present, there are few studies on circABCC4, mainly focusing on cancer (Huang et al., 2019). Studies have found that circABCC4 is highly expressed in lung adenocarcinoma and prostate cancer (Huang et al., 2019; Liu et al., 2020). Multidrug resistance-related protein 4 (MRP4) is a member of ABCC. Inhibition of MRP4 can significantly reduce sepsis-induced ALI (Xia et al., 2019).

Arachidonic acid metabolites may be involved in the process of lung injury. A key enzyme regulating the metabolism of arachidonic acid is cytosolic phospholipase A2 (cPLA2), which is encoded by PLA2G6 (Bellido-Reyes et al., 2006). PLA2G6 is highly expressed in lung injury and inflammatory diseases. Leukotrienes B4 (LTB4) has been reported to be chemotactic through recruiting inflammatory cells to the lungs (Ford-Hutchinson et al., 1980). Studies have also shown that the LTB4/LTB4R pathway is involved in the pathogenesis of human inflammatory diseases of lung (Wilson et al., 2014). Klebs von den Lungen-6 (KL-6) is an efficient predictor of interstitial lung disease (Jiang et al., 2018). Interstitial lung disease is mainly caused by type II lung epithelial cell injury and abnormal accumulation of extracellular matrix in lung parenchyma. Similar pathology is also seen in BPD (Wang et al., 2014). It has been reported that KL-6 level in peripheral blood is closely related to the severity of BPD (Wang et al., 2014; Matsumura et al., 2017). Superoxide dismutase (SOD) is required for repairing neovascularization in response to ischemic injury by preventing excessive production of superoxide (Kim et al., 2007). In many BPD models, the expression and activity of SOD in the lung show abnormality (Ahmed et al., 2003).

Through genetic screening, we found that circABCC4 is related to BPD. Bioinformatics analysis and preliminary experiments found that circABCC4 is related to miR-663a, and that miR-663a is down-regulated and the downstream target gene PL2G6 is up-regulated. However, the specific mechanism is unknown, so this study intends to further explore whether circRNA circABCC4 promotes BPD progression via facilitating PLA2G6 expression by sequestering miR-663a.

## Materials and Methods

### Subjects and Sample Collection

From September 2019 to November 2019, we enrolled a total of 62 infants with BPD and unrelated healthy controls in the First Affiliated Hospital of Nanjing Medical University. After the parents’ informed consent, peripheral blood samples were obtained from the subjects and the control group (Table 1). The study was approved by the Clinical Research Ethics Committee of Nanjing Medical University (2016-SR-155).

**TABLE 1:** Characteristics of enrolled infants.

	Control group (*n* =31)	BPD group (*n* =31)
Birth weights (grams)*	2,311 ± 593	1,664 ± 669
Gestational age (weeks)*	34 ± 2	30 ± 3
**Sex**		
Male	12 (39%)	17 (55%)
Female	19 (61%)	14 (45%)
Antenatal steroids	14 (45%)	20 (65%)
Cesarean section	20 (65%)	21 (68%)
Vaginal	11 (35%)	10 (32%)
**Apgar score**		
1 min		
=3	0 (0%)	3 (10%)
4–6	2 (6%)	6 (20%)
>6	29 (94%)	22 (70%)
** 5 min**		
=3	0 (0%)	2 (6%)
4–6	0 (0%)	2 (6%)
>6	31 (100%)	27 (88%)

*Mean ± SD.

### Cell Culture and Hyperoxia Exposure

Human type II alveolar lung epithelial cells (A549) cells and HEK293 cell lines were purchased from Shanghai Institutes for Biological Sciences. Cells were maintained in a 5% CO_2_ incubator at 37°C in Dulbecco’s Modified Eagle Medium (DMEM) supplemented with 10% FBS and penicillin/streptomycin (100 U/ml). A549 cells were randomly divided into two groups: the control group and the hyperoxia group. The control group was exposed to normoxic gas (21% O_2_/5% CO_2_/74% N_2_). For high oxygen exposure, cells were placed in a high oxygen incubator filled with 85% O_2_ and 5% CO_2_.

### Animals and Study Design

There were 50 newborn Sprague-Dawley (SD) rats, including 20 males (220–250 g) and 30 females (200–220 g), which were purchased from Nanjing Medical University (Nanjing, China). Within 12 h of birth, newborn SD rats were randomly divided into two litters and continuously exposed to either 21% O_2_ (room air) and/or 85% O_2_ (hyperoxia) for 1, 3, 7, 14, and 21 days. The temperature (25°C) and humidity (50–60%) in the feeding box were kept constant. The GT-903 oxygen concentration detector (Korno company, Shenzhen, China) was used to monitor oxygen concentration, ensuring that newborn rats were exposed to 85% O_2_, while the other half of newborn rats were exposed to 21% O_2_ indoor air. At the ages of 1, 3, 7, 14, and 21 days, young rats were killed to obtain lung tissue. The experimental protocol was approved by the Committee on the Ethics of Animal Experiments of The First Affiliated Hospital with Nanjing Medical University (permit number: IACUC-1812026).

### Bioinformatics Analysis

circRNA and miRNA were selected by the Agilent circRNA chip (Shanghai Biotechnology Corporation). Bioinformatics tool TargetScan (Release7.2)^1^ predicted downstream target genes of miR-663a. The results indicated that that the 3′-UTR of PLA2G6 binds to miR-663a with a high score, suggesting PLA2G6 might be a target of miR-663a.

### Plasmid and siRNA

MiR-663a-mimic, miR-663a-inhibitor and the corresponding negative control were synthesized by RiboBio. The double-stranded siRNA and siNC were synthesized and high performance purified (GenePharma). The targeted and negative control siRNA for PLA2G6 mRNA were as follows: PLA2G6: 5′-GCCUAAACAACCUAGAACUTT-3′ (sense); Control: 5′-UUCUCCGAACGUGUCACGUTT-3′ (sense). The wild type (WT) and mutated (MUT) 3′-UTR sequences of PLA2G6 were purchased from Tsingke (Nanjing, China), which were predicted to interact with miR-663a, were cloned into a firefly luciferase-expressing pmiR-GLO vector (Tsingke) to acquire the PLA2G6 3′-UTR reporter constructs (pmiR-PLA2G6-WT and pmiR-PLA2G6-MUT).

### Cell Transfection

A549 cells cultured in six-well plate were transfected with 3 μl of mimic or 5 μl of inhibitor with miR-663a by Lipofectamine 3000 (Invitrogen, Thermo Fisher Scientific). Twenty four hours after transfection, the fresh medium was replaced. Next, the A549 cells were treated with high oxygen for 72 h. Lentivirus packaging cells were transfected with hU6-MCS-Ubiquitin-EGFP-IRES-puromycin vector (GENECHEM, China) containing either the circABCC4 knockdown (sh-circABCC4:5′-GAAGAATCCAGGAGCGGAC-3′), circABCC4 overexpression (LV- circABCC4), PLA2G6 overexpression (LV-PLA2G6) and a negative control sequence (NC), respectively. Lentiviral transduction was performed in A549 cell lines. Pools of stable transductants were generated by selection using puromycin (4 μg/ml) for 2 weeks.

### Nuclear-Cytoplasmic Fractionation and RNase R Treatment

Nuclear and cytoplasmic RNA were isolated with the PARIS^TM^ kit (Invitrogen, United States) as directed by the manufacturer. For RNase R treatment, 2.5 μg total RNA was incubated for 30 min at 37°C with 10 U RNase R (Guangzhou Geneseed Biotech Co, China).

### RNA Fluorescence *in situ* Hybridization (FISH)

The Cy3-labeled circABCC4 probe was designed and synthesized by Riobo (Guangzhou, China). The DIG-labeled circABCC4 specific probe was hybridized with the cells overnight in hybridization buffer.

### RNA Immunoprecipitation (RIP) Assay

RIP experiment was conducted using the Magna RIP^TM^ RNA-Binding Protein Immunoprecipitation Kit (Millipore, Billerica, MA, United States). A549 cells were transfected with miR-663a mimic or negative control. A549 cells are lysed under hyperoxic environment. Then, the RIP immunoprecipitation buffer including magnetic beads conjugated with negative control rabbit IgG or human anti-AGO2 antibody (Rabbit, Millipore, Billerica, United States) was added into cell lysates. Subsequently, the lysates were rotated overnight. Next day, after incubating with Proteinase K for 30 min, the immunoprecipitated RNA was extracted. Last, qRT-PCR was performed to identify the expression of circABCC4 and miR-663a.

### Luciferase Reporter Assays

A549 cells and HEK-293 cells were seeded into 96-well plates 24 h before transfection. MiR-663a mimic (30 nM) and miR-663a mimic NC (30 nM) and miR-663a inhibitor (50 nM) and inhibitor NC (50 nM) were transfected into A549 or HEK-293 cells by using Lipofectamine 3000 according to the manufacturer’s instructions. For luciferase reporter assays, the 3′UTR of PLA2G6 gene contains miR-663a binding site (CCCCGCC) was cloned. PLA2G6-WT-Reporter plasmid containing wildtype 3′UTR of PLA2G6, and PLA2G6-Mut-Reporter plasmid containing mutant-type 3′UTR of PLA2G6 were synthesized. Subsequently, the cells were cotransfected with 1,000 ng of each of the PLA2G6-WT-Reporter plasmid and PLA2G6-Mut-Reporter plasmid, together with miR-663a mimic, miR-663a mimic NC, miR-663a inhibitor, or miR-663a inhibitor NC. 24 h after transfection, cells were lysed and luciferase activity was determined using the Dual -Luciferase Reporter Assay System (Promega). All results from at least three independent experiments.

### RNA Extraction and Quantitative Real-Time PCR (qRT-PCR)

Total RNA samples from blood samples of infants, A549 cells and animal tissues were extracted separately using TRIzol reagent (Invitrogen, United States) according to the manufacturer’s protocol. For circRNA and mRNA, RNA was reversely transcribed into cDNA using a PrimeScript^TM^ RT Master Mix reagent kit (TaKaRa, Shiga, Japan). For miRNA, cDNA was synthesized by the PrimeScript^TM^ RT reagent kit (RiboBio, GuangZhou, China). qRT-PCR was performed in the LightCycler480II (Roche) using SYBR Green technology (Takara). The relative expression level was calculated by comparing the 2^–ΔΔCt^ method. The primers for miR-663a and U6 were purchased from RiboBio (RiboBio, Guangzhou, China). The primers for circABCC4 was purchased from GENECHEM (Shanghai, China). The primers for GAPDH, PLA2G6, LTB4R, KL-6, and SOD were purchased from Tsingke (Nanjing, China):

GAPDH:

Forward: 5′-CGCTCTCTGCTCCTCCTGTT-3′,

Reserve: 5′-CATGGGTGGAATCATATTGG-3′,

PLA2G6:

Forward: 5′-AACCAGATCCACAGCAAGGAT-3′,

Reserve: 5′-GTGTTCCCATGCTCTCCTCG-3′,

LTB4R:

Forward: 5′-AGCTTTGTGGTGTGGAGTATCC-3′,

Reserve: 5′-GCAACCAGCCAGTCCAAAAC-3′,

KL-6:

Forward: 5′-CAACCCCAGCCAGCAAGAG-3′,

Reserve: 5′-AAAGACAGGAAAAAGAAAGAGACCC-3′,

SOD:

Forward: 5′-AACCAGTTGTGGTGTCAGGA-3′,

Reserve: 5′-CATTGCCCAGGTCTCCAACA-3′.

### Western Blot

Total protein from rat lung tissues or cells was extracted using RIPA lysis buffer (Beyotime). Equal amount of protein was separated by 10% SDS-PAGE gels and blotted on a PVDF membrane (Millipore). To block non-specific sites, the membranes were incubated in 5% dry milk in TBS-T saline for 2 h, the membrane immunodetected with anti-PLA2G6 (abcam), anti-LTB4R (abcam), anti-KL-6 (Bioss), anti-SOD (abcam), and anti-β-actin antibody (Proteintech) at 4°C overnight. Then membranes were washed three times with TBS-T and treated with goat anti-rabbit IgG (Proteintech) or goat anti-mouse IgG (Proteintech). Signals of membranes were measured by chemiluminescence (ECL) system, scanned and analyzed by Image Lab Software (Bio-Rad).

### Cell Counting Kit-8 (CCK8) Proliferation Assay

Cell viability was measured using CCK-8 proliferation assay (DOjindo, Japan). Cells were seeded at a density of 3 × 10^3^ cells/well in 96-well plates with three replicates. Following incubation for 72 h, CCK-8 solution (10 μL/well) was added to each well. After incubation for 2 h, the optical density (OD) value of cck-8 was measured at 450 nm test wavelength with a microplate reader (Thermo Fisher Scientific, United States).

### EdU Assay of Proliferation

EdU analysis was performed using Cell-Light EdU DNA cell proliferation kit (RiboBio, Guangzhou, China). Cells (1 × 10^4^) were seeded in each well of a 96-well plate. After incubating with 50 μM EdU for 2 h, the cells were fixed in 4% paraformaldehyde and stained with Apollo dye solution. Images were obtained with a Nikon microscope (Nikon ECLIPSE Ti, Japan).

### Lung Histology and Morphometric Analysis

After 1, 3, 7, 14, and 21 days of continuous exposure to hyperoxia or indoor air, premature rats were sacrificed by intraperitoneal injection of chloral hydrate (100 mg/kg). The lungs were removed, immersed in a paraformaldehyde solution overnight, and the right lower lobe was embedded in paraffin. The sections were then dissected and mounted on RNase-free slides. Rat lung tissues were stained with haematoxylin and eosin (H&E). Morphological analysis was performed on at least three lung sections of each animal. Alveolarization was assessed by performing radial alveolar counting (RACs). From the center of the respiratory bronchioles, draw vertically to the connective tissue septum or pleural defined acinar edge and count the number of septa that intersect the line. Each animal was counted a total of 10 times.

### Immunohistochemistry (IHC)

Immunohistochemical staining (CWBIO, China) was performed on the tissues on 1, 3, 7, 14, and 21 days after hyperoxia or air exposure. After dewaxing, slide the slide with PLA2G6 antibody (1:400, Bioss), LTB4R antibody (1:400, Bioss), KL-6 antibody (1:400, Bioss), or SOD antibody (1:200, Abcam) Incubate at 4°C overnight, then wash three times with PBS for 5 min each. Sections were then incubated with a 500-fold diluted secondary antibody for 1 h. Finally, the slides were viewed under a microscope (Nikon ECLIPSE Ti, Japan).

### Detection of Apoptosis

According to the manufacturer’s instructions (Thermo), apoptosis was detected using the TUNEL assay after 1, 3, 7, 14, and 21 days of high oxygen or air exposure. Briefly, the sections were paraffin-free, digested with proteinase K (1:200) at 37°C for 10 min, and then soaked in PBS for 5 min. Each section was covered with a terminal deoxynucleotide transferase (TDT) enzyme solution and incubated in a wet chamber at 37°C for 2 h. The sections were immersed in the termination buffer to terminate the enzymatic reaction, rinsed gently with PBS. Sections were stained with DAB for 10 min and observed under a microscope (Nikon ECLIPSE Ti, Japan). Sections incubated with PBS instead of TDT enzyme solution were used as negative controls. Tunel-positive cells were counted at the same magnification in six randomly selected fields in control and treatment mice (magnification, x400).

### Flow Cytometry

Apoptosis assay was carried out using the Annexin V-FITC/PI apoptosis kit (Lianke, China) according to the manufacturer’s protocol. Briefly, the cells were collected after treated for 72 h, and washed twice with cold PBS, and then resuspend cells in 1 × Binding Buffer at a concentration of 1 × 10^6^ cells/ml. Subsequently, 5 μl of Annexin V-FITC or 10 μl of 7-PI was added to the cell suspension and incubated for 5 min at room temperature in the dark and then analyzed by flow cytometry (BD, United States).

### Statistical Analysis

All experiments were repeated at least 3 times. The results were presented as the mean ± standard error. All statistical analyses were performed using SPSS 22.0 (IBM, SPSS, Chicago, IL, United States) and GraphPad Prism version 7.0 software; *P* < 0.05 was considered statistically significant. Genes with a fold change of ≥ 2 in sequencing or microarray data and a *p* < 0.05 were considered to have significant differential expression.

## Results

### circRNA Expression Profiles in BPD

**FIGURE 1:**
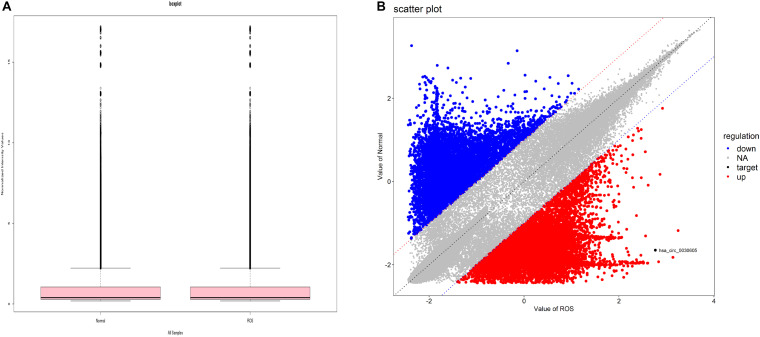
circRNA expression profile in BPD. **(A)** Boxplot, a method of describing data in terms of minimum, first quartile (25%), median (50%), third quartile (75%), and maximum. the abscissa is the sample name, and the ordinate is the value of the signal value of the probe after log2. The upper and lower sides of the rectangular box correspond to the upper and lower quartiles of the data (Q1 and Q3). The median line inside the rectangular box is the median, the upper line is Q1+1.5IQR, and the underline is Q3-1.5IQR, where IQR (Inter) Quantile Range) is the middle quartile range, IQR = Q3-Q1. **(B)** Each dot in the scatter plot represents the probe point on the chip. The *x*-axis represents the signal value after this point is normalized in the control chip. The *y*-axis represents the signal value after the point is normalized in the sample chip. The point on the y = x line (the median line on the graph) in the graph, which represents the probe point Fold Change = 1 in both chips. A point that falls outside the 45° line on either side of the bit line in the graph, which represents the probe point Fold Change > 2 in both chips. The red circles in the plot represent the upregulated circRNAs, and the blue circles in the plot represent the downregulated differentially expressed circRNAs with statistical significance.

Through bioinformatics analysis, the circRNA expression profile in BPD patients and normal peripheral blood was revealed. Box plots (Shanghai Biotechnology Corporation) were used to show the data symmetry, the degree of dispersion (Figure 1A), and scatter plots to show the changes of circRNA expression in BPD patients and the normal population (Figure 1B).

**FIGURE 2:**
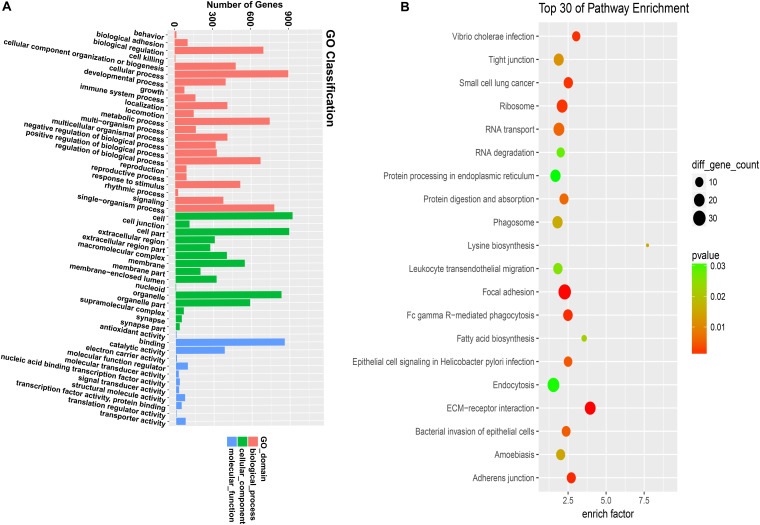
GO and KEGG analyze the gene function of differentially expressed circular RNA. **(A)** GO analysis covers the three domains of cellular component, molecular function, and biological process. The *x*-axis represents the number and proportion of all annotated genes for each term function, and the *y*-axis represents the number and proportion of all annotated genes. **(B)** KEGG analysis revealed the most important biochemical metabolic pathways and signal transduction pathways involved in gene involvement. The *x*-axis represents the enrich factors in the pathway, the *y*-axis represents the relevant pathway, the *p*-value is represented by a different color, and the size of the circle represents the number of genes in the pathway.

According to the corresponding relationship between circRNA and its parental gene, GO and KEGG enrichment analyses were conducted to determine the function of the parental gene of the differentially expressed circRNA (Figure 2A). The figure shows 30 the most significant enrichment pathways (Figure 2B).

### Expression Profile of CircABCC4, miR-663a, and PLA2G6 of BPD Model *in vitro*

**FIGURE 3:**
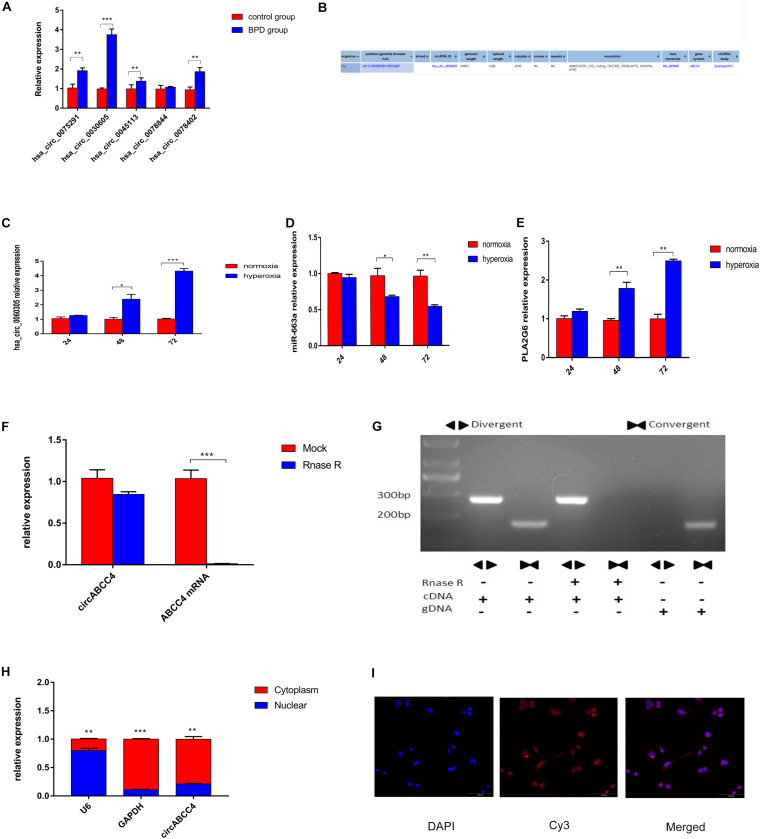
CircABCC4 is up-regulated in BPD and correlated with the progression. **(A)** Relative expression of circRNAs in control group or BPD group by qRT-PCR. **(B)** According to the circBase annotation, hsa_circ_0030605 is also known as circABCC4. **(C–E)** Relative expression of circABCC4,miR-663a or PLA2G6 in normoxia or hyperoxia by qRT-PCR. **(F)** qRT-PCR analysis of circABCC4 and linear ABCC4 abundance in A549 cell line after RNase R treatment. **(G)** The convergent and divergent primers were used to amplify the linear and anti-splice products, whether or not they were treated with RNase R, and subjected to polymerase chain reaction. **(H)** qRT-PCR analysis confirmed that circABCC4 and ABCC4 were mainly located in the cytoplasm of the A549 cell line. **(I)** RNA fluorescence *in situ* hybridization for circABCC4. Nuclei were stained with 4,6-diamidino-2-phenylindole (DAPI). Scale bar, 100 μm. All data are presented as the mean ± SEM of three experiments. *P < 0.05, **P < 0.01, ***P < 0.001.

According to the sequencing results, the expression level of circABCC4 (has_circ_0030605) in BPD was up-regulated. We screened the top five circRNAs with different multiples for verification by RT-qPCR. The results showed that circABCC4 expression was up-regulated under BPD group, compared with those in control group. Although its fold change was not the largest, the circABCC4 expression showed the strongest relevance to BPD among all the circRNAs (Figure 3A). According to the sequencing results and the circbase^2^, we presume that circABCC4 has a genomic length of 94,902 nt and a spliced length of 1,280 nt. Its location in the genome is chr13:95858785-95953687 (Figure 3B). In addition, miR-663a was down-regulated, circABCC4 (has_circ_0030605), and PLA2G6 was up-regulated under hyperoxic exposure, compared to that in normoxic condition (Figures 3C–E).

### Characterization of CircABCC4 in A549 Cell

In A549 cell lines, mRNA expression was decreased due to digestion by RNase R, but circRNA expression was not influenced by RNase R (Figure 3F). The stability of circABCC4 was detected by PCR using cDNA and gDNA (genomic DNA) pretreated or not treated with RNase R (Figure 3G). Under RNase R treatment, circABCC4 was resistant to digestion, while the linear form of ABCC4 was rapidly digested. Compared with cDNA, gDNA was not found to have a circRNA amplification product.

In terms of localization, qRT-PCR analysis was performed after physical separation of nucleoli and cytoplasmic RNA. Its results showed that circABCC4 was preferentially localized in the cytoplasm (Figure 3H). Fluorescence *in situ* hybridization (FISH) also showed that circABCC4 was preferentially localized in the cytoplasm (Figure 3I). Therefore, our results indicated that circABCC4 is a stable cytoplasmic circRNA. This implies that circABCC4 has the potential to be a diagnostic marker for BPD.

### Progression of BPD Promoted by CircABCC4 via Up-Regulating PLA2G6

**FIGURE 4:**
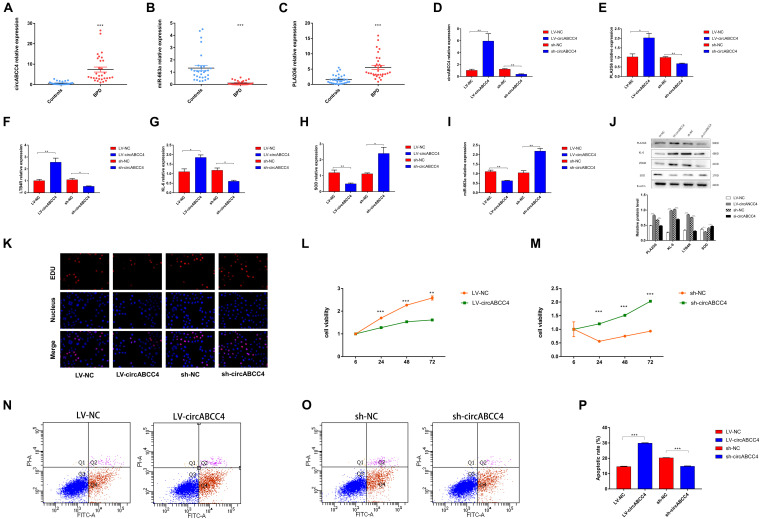
circABCC4 is upregulated in BPD and associated with clinical parameters. **(A)** qRT-PCR analysis of peripheral blood in 31 BPD patients and 31 non-lung-infected neonates revealed that circABCC4 expression was up-regulated in BPD. **(B,C)** The qRT-PCR analysis of miR-663a in peripheral blood of 31 BPD patients and 31 non-lung-infected newborns was significantly down-regulated in BPD samples. The expression of PLA2G6 was up-regulated. **(D–I)** Relative expression of circABCC4,miR-663a, PLA2G6, LTB4R, KL-6, and SOD mRNA was detected by qRT-PCR in A549 cells after transfection of sh-circABCC4, LV-circABCC4 or negative control. **(J)** Western blotting of PLA2G6, KL-6, LTB4R, and SOD in A549 cells transfected with sh-circABCC4, LV-circABCC4 or negative control. Protein levels have been quantified. **(K)** Assessment of DNA synthesis using an EdU (5-ethynyl-2′-deox-yuridine) assay in A549 cells transfected with negative control, LV-circABCC4 or sh-circABCC4. Micrographs represent at least three experiments. Scale bar, 50 μm. **(L,M)** CCK8 assay of cells transfected with sh-circABCC4, LV-circABCC4 or negative control. **(N,O)** Transfection of LV-circABCC4, sh-circABCC4 and negative control combined with PI/AV-FITC to detect apoptosis. **(P)** Flow cytometry is used to assess the rate of apoptosis. All data are presented as the mean ± SEM of three experiments. ^∗^*P* < 0.05, ^∗∗^*P* < 0.01, ^∗∗∗^*P* < 0.001.

Because circABCC4 is mainly localized in the cytoplasm, we hypothesized that circABCC4 can be used as a miRNA sponge to increase the expression of target genes. The microarray results showed that circABCC4 was up-regulated in patients with BPD. To determine the level of circABCC4, we collected peripheral blood from 62 BPD neonates and control neonates without lung infection for qRT-PCR, and the results were consistent with the microarray results. However, miR-663a was down-regulated and the downstream target genePLA2G6 was up-regulated (Figures 4A–C). After the down-regulation of circABCC4 in the cell line, the expression of PLA2G6, LTB4R, and KL-6 in A549 cells were significantly decreased, while that of miR-663a and SOD was increased. This is because SOD overexpression can reverse the lung injury caused by high-level oxygen (Buczynski et al., 2018). However, when circABCC4 expression was increased, the opposite result was obtained (Figures 4D–I). Therefore, we assume that circABCC4 positively regulates the expression of PLA2G6. In addition, the down-regulation or up-regulation of circABCC4 reduced or increased the expression of PLA2G6, LTB4R, KL-6, or SOD protein in A549 cells under hyperoxic condition, respectively (Figure 4J).

We performed a 5-ethynyl-2′-deoxyuridine (EdU) incorporation assay to detect A549 cell proliferation. After up-regulation of circABCC4, the number of EdU+ cells was significantly decreased compared with that in control cells. The results were reversed after knocking-down circABCC4 (Figure 4K). In addition, we also conducted the CCK-8 test, and the results were consistent with the results obtained by the EDU assay (Figures 4L,M). To examine the effect of circABCC4 on cell function, we conducted apoptosis experiments. The results showed that the overexpression of circABCC4 increased apoptosis. The down-regulation of circABCC4 inhibited cell apoptosis (Figures 4N–P).

### CircABCC4 Acting as a Sponge of miR-663a

**FIGURE 5:**
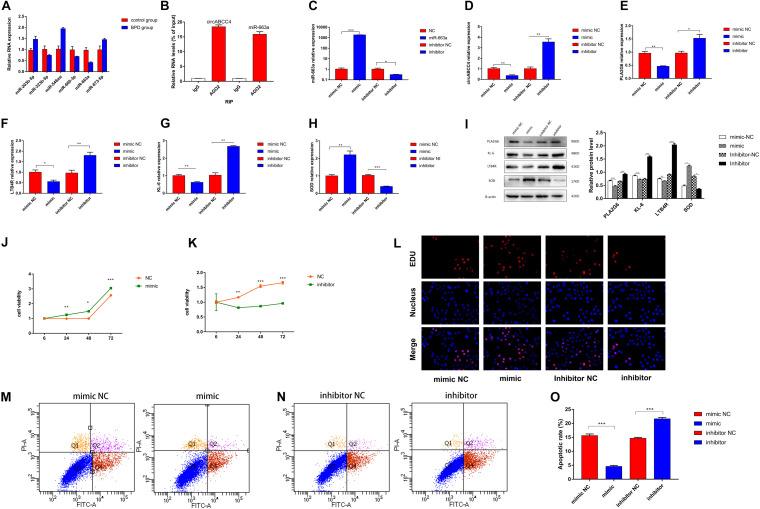
CircABCC4 promotes BPD progression by serving as a miRNA sponge of miR-663a. **(A)** Relative expression of miRNAs in control group or BPD group by qRT-PCR. **(B)** RNA immunoprecipitation (RIP) assay results confirmed that Argonaute 2 (Ago2) immunoprecipitated with both circABCC4 and miR-663a in A549 cells. **(C–H)** Relative expression of miR-663a, circABCC4, PLA2G6, LTB4R, KL-6, and SOD mRNA was detected by qRT-PCR in A549 cells after transfection of mimic, inhibitor or negative control. **(I)** Western blotting of PLA2G6, KL-6, LTB4R, and SOD in A549 cells transfected with mimic, inhibitor or negative control. Protein levels have been quantified. **(J,K)** CCK8 assay of cells transfected with mimic, inhibitor or negative control. **(L)** Assessment of DNA synthesis using an EdU (5-ethynyl-2′-deox-yuridine) assay inA549 cells transfected with negative control, mimic or inhibitor. Micrographs represent at least three experiments. Scale bar, 50 μm. **(M,N)** Transfection of mimic, inhibitor and negative control combined with PI/AV-FITC to detect apoptosis. **(O)** Flow cytometry is used to assess the rate of apoptosis. All data are presented as the mean ± SEM of three experiments. *P < 0.05, **P < 0.01, ***P < 0.001.

More and more studies have reported that circRNA can act as a miRNA sponge. Therefore, we investigated whether circABCC4 has the ability to bind miRNA. Through the microarray results, we found that 76 miRNAs may be targets of circABCC4. We screened the top 6 miRNAs with differential expression multiples for verification. According to the mechanism of ceRNA, we finally determined that miR-663a is one of the key genes related to circABCC4 (Figure 5A). In the study, we investigated whether circABCC4 promotes the progress of BPD through sponging miRNAs. Therefore, A549 cells were subjected to anti-AGO2 RIP assay in hyperoxic environment, and anti-AGO2 antibody (IgG as a negative control, none as input) was used to pull down circABCC4 and miR-663a. The results of RIP experiments showed that in A549 cells, circABCC4 and miR-663a were effectively pulled down by anti-AGO2 antibodies, not by non-specific anti-IgG antibodies (Figure 5B).

In order to verify the efficiency of A549 cells transfected with miR-663a mimics, inhibitors and NC under hyperoxic condition, the relative expression of miR-663a was verified by real-time fluorescence quantitative PCR. MiR-663a mimic transfection significantly increased the expression of miR-663a in cells compared with mimic-NC transfection (Figure 5C). Compared with inhibitor NC, miR-663a inhibitor significantly reduced the expression of miR-663a (*p* < 0.001). Then, in order to determine whether the synthesis of upstream circRNA and downstream target gene is regulated by miR-663a in A549 cells under hyperoxia, the relative expression levels of circABCC4 and PLA2G6 were detected in further experiments (Figures 5D,E). At the same time, after intervention of miR-663a under hyperoxia, the expression of LTB4R, KL-6, and SOD, was measured (Figures 5F–H). According to the results of RT-PCR, the mRNA levels of circABCC4, PLA2G6, LTB4R, and KL-6 in A549 cells after the overexpression of miR-663a was decreased. However, the mRNA level of SOD in A549 cells was increased after the over-expression of miR-663a. Compared with the negative control, the down-regulation of miR-663a exerted the opposite effect on the expression of LTB4R, KL-6, and SOD in A549 cells under hyperoxia.

We used Western blot analysis to determine this effect at the protein level. Compared to negative control cells, overexpression of miR-663a reduced the expression of protein levels of PLA2G6, LTB4R, and KL-6, but increased the expression of SOD. Inhibition of miR-663a increased the expression of PLA2G6, LTB4R, and KL-6, and decreased the expression of SOD (Figure 5I).

### Reversed Ability of CircABCC4 and PLA2G6 by miR-663a to Promote the Progress of BPD

To determine the effect of miR-663a on the proliferation of A549 cells exposed to hyperoxia, we performed a CCK-8 assay. The results showed that miR-663a overexpression promoted cell proliferation. In contrast, knockdown of miR-663a brought with the opposite result (Figures 5J,K). EDU assay results were consistent with CCK-8 results (Figure 5L). Apoptosis experiments showed that miR-663a mimic inhibits apoptosis, while miR-663a inhibitors promote apoptosis (Figures 5M–O).

### PLA2G6 as a Direct Target of miR-663a

**FIGURE 6:**
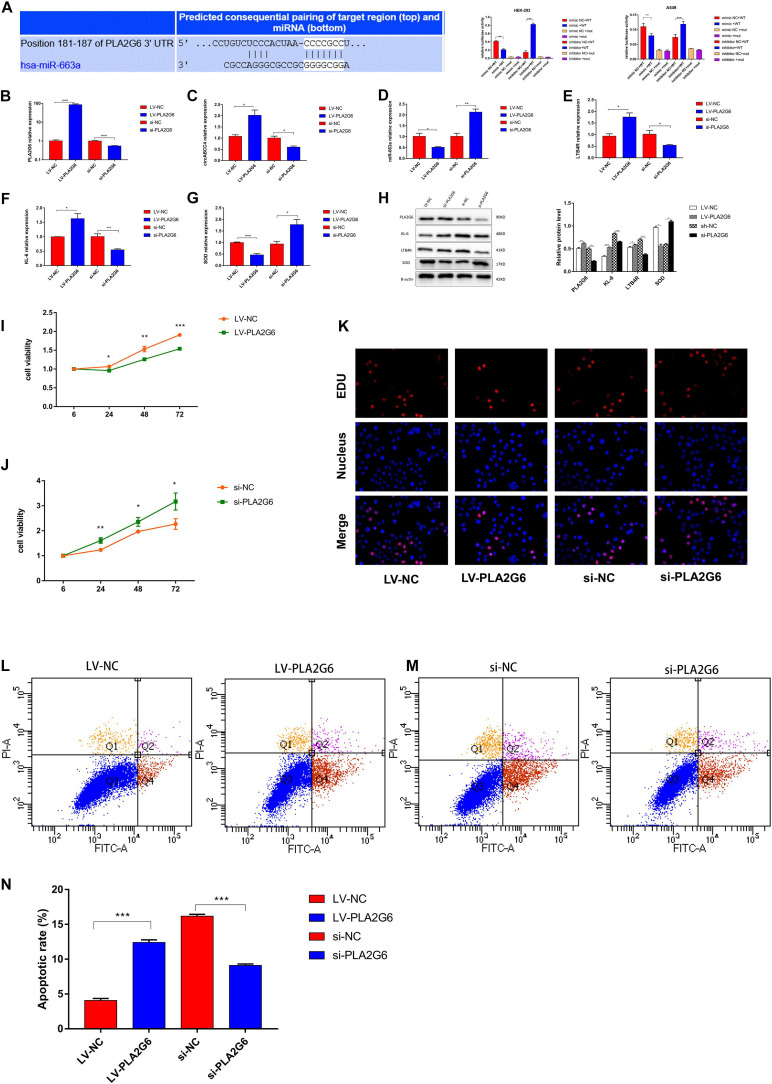
MiR-663a suppresses BPD progression through directly targeting PLA2G6. **(A)** The putative-binding site of miR-663a with PLA2G6 predicted by TargetScan. A luciferase reporter assay was used to detect the binding of miR-663a with PLA2G6-3’UTR and Mut-PLA2G6-3’UTR. **(B–G)** Relative expression of PLA2G6, circABCC4, miR-663a, LTB4R, KL-6, and SOD mRNA was detected by qRT-PCR in A549 cells after transfection of LV-PLA2G6, si-PLA2G6 or negative control. **(H)** Western blotting of PLA2G6, KL-6, LTB4R, and SOD in A549 cells transfected with LV-PLA2G6, si-PLA2G6 or negative control. Protein levels have been quantified. **(I,J)** CCK8 assay of cells transfected with LV-PLA2G6, si-PLA2G6 or negative control. **(K)** Assessment of DNA synthesis using an EdU (5-ethynyl-2′-deox-yuridine) assay inA549 cells transfected with negative control, LV-PLA2G6 or si-PLA2G6. Micrographs represent at least three experiments. Scale bar, 50 μm. **(L,M)** Transfection of LV-PLA2G6, si-PLA2G6 or negative control combined with PI/AV-FITC to detect apoptosis. **(N)** Flow cytometry is used to assess the rate of apoptosis. All data are presented as the mean ± SEM of three experiments. *P < 0.05, **P < 0.01, ***P < 0.001.

The TargetScan database shows that the PLA2G6 mRNA contains the MRE of miR-663a, which means that miR-663a may directly target PLA2G6 (Figure 6A). Then, we used hsa_circ_0030605, 57 miRNAs and 14 target genes, and constructed a hsa_circ_0030605-miRNA-target gene network by using Cytoscape to visualize their interaction (Supplementary Figure S1A). Luciferase reporter assay showed that miR-663a mimic significantly reduced luciferase activity of WT in A549 and HEK-293 cells, whereas miR-663a inhibitor significantly increased luciferase activity of WT, while mutants showed no change in luciferase activity (Figure 6A). The putative binding site of miR-663a relative to PLA2G6 was determined by StarBase v3.0 (Supplementary Figure S1B). These data indicate that miR-663a inhibits the expression of PLA2G6 by directly binding to the 3′-UTR of PLA2G.

To verify the efficiency of A549 cells transfected with PLA2G6 overexpressing lentivirus or siRNA under hyperoxic conditions, the relative expression of PLA2G6 was verified by RT-PCR (Figure 6B). The over-expression of PLA2G6 significantly increased the expression of circABCC4 in A549 cells, while decreased the expression of miR-663a. Knocking down PLA2G6 in A549 cells at high oxygen levels had the opposite effect (Figures 6C,D). Secondly, PLA2G6 was overexpressed in A549 cells under hyperoxic conditions, and the relative expressions of LTB4R and KL-6 were increased, while the expression of SOD was decreased, compared with the negative control group. Knocking down PLA2G6 gave the opposite result (Figures 6E–G).

To confirm this effect at the protein level, we collected A549 cells that were overexpressed or knocked down PLA2G6 for western blot analysis. After the over-expression of PLA2G6, protein levels of LTB4R and KL-6 increased, while the protein level of SOD decreased. When PLA2G6 siRNA was transfected into A549 cells under hyperoxic condition, the opposite result was obtained (Figure 6H).

### Effect of PLA2G6 Interference in A549 Cell Line on the Development of BPD

To examine the effect of PLA2G6 on cell function, we conducted CCK-8, EDU and flow cytometry. The results showed that the overexpression of PLA2G6 inhibited cell proliferation and increased apoptosis. The knockdown of PLA2G6 increased cell proliferation and decreased cell apoptosis (Figures 6I–N).

### PLA2G6 Expression Rescued and A549 Cell Proliferation Inhibited by CircABCC4 Overexpression

**FIGURE 7:**
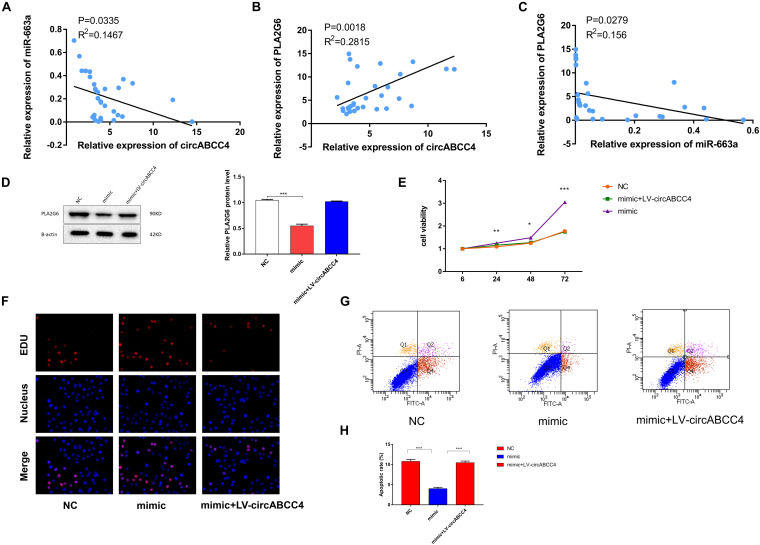
Overexpression of circABCC4 reversed PLA2G6 expression and proliferation, which were inhibited by miR-663a. **(A)** Pearson correlation analysis determined the significantly negative correlation between the levels of circABCC4 and miR-663a in BPD patients and neonates without pulmonary infection. **(B)** The level of PLA2G6 positively correlated with the level of circABCC4 in BPD patients and neonates without pulmonary infection by Pearson correlation analysis. **(C)** Pearson correlation analysis determined the significantly negative correlation between the levels of PLA2G6 and miR-663a in BPD patients and neonates without pulmonary infection. **(D)** Western blot analysis of PLA2G6 with proteins treated with miR-663a mimics and circABCC4 overexpression lentivirus in A549 cell line. Protein levels have been quantified. **(E)** Assessment of proliferation of A549 cell transfected with negative control, miR-663a mimics, or circABCC4 overexpression lentivirus by CCK-8. **(F)** Assessment of DNA synthesis using an EdU (5-ethynyl-2′-deox-yuridine) assay inA549 cells transfected with negative control, mimic or mimic+LV-circABCC4. Micrographs represent at least three experiments. Scale bar, 50 μm. **(G)** Transfection of mimic, mimic+LV-circABCC4 or negative control combined with PI/AV-FITC to detect apoptosis. **(H)** Flow cytometry is used to assess the rate of apoptosis. All data are presented as the mean ± SEM of three experiments. ^∗^*P* < 0.05, ^∗∗^*P* < 0.01, ^∗∗∗^*P* < 0.001.

Next, we conducted a rescue experiment to determine whether circABCC4 promoted the biological function of BPD progression through the circABCC4-miR-663a- PLA2G6 axis. We found that the expression of PLA2G6 and circABCC4 in peripheral blood of BPD patients were higher than those in patients without pulmonary infection, while the results of miR-663a were reversed. Linear correlation analysis was performed on circABCC4 and miR-663a or PLA2G6. The results showed that circABCC4 was negatively correlated with miR-663a (*P* = 0.0335, *R*^2^ = 0.1467, Figure 7A) and positively correlated with PLA2G6 (*P* = 0.0003, *R*^2^ = 0.2815, Figure 7B), and PLA2G6 was negatively correlated with miR-663a (*P* = 0.0279, *R*^2^ = 0.156, Figure 7C), which strongly suggests the involvement of the circABCC4-miR-663a-PLA2G6 axis in BPD. Next, to further elucidate this axis, the A549 cell line was co-transfected with miR-663a mimic and circABCC4 overexpression lentivirus, and the protein expression of PLA2G6 was detected. Western blot confirmed that the protein expression level of PLA2G6 was significantly down-regulated after treatment with miR-663a mimic. Overexpression of circABCC4 rescued the expression of this protein (Figure 7D). CCK-8 and EDU assay results showed that miR-663a mimic alone significantly increased cell growth. This effect could be rescued by co-transfection with miR-663a mimic and circABCC4 overexpression lentivirus in the A549 cell line, confirming that the cell proliferation is regulated by the circABCC4-miR-663a-PLA2G6 axis (Figures 7E,F). At the same time, the results of flow cytometry also meet this conclusion (Figures 7G,H).

### Neonatal Rat Growth and Pulmonary Histology

**FIGURE 8:**
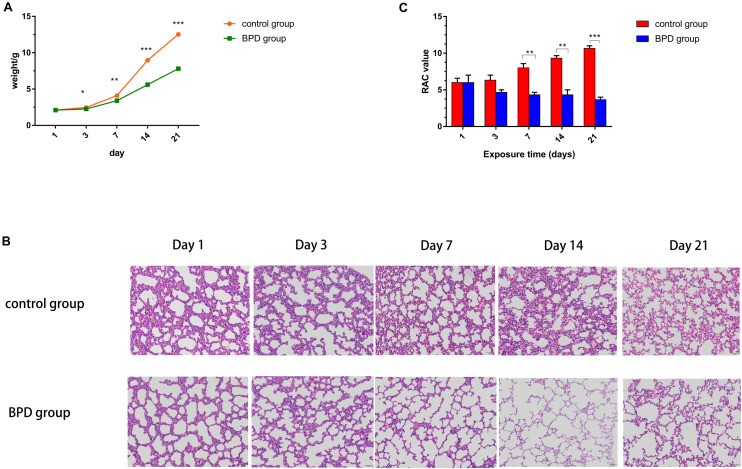
Establishment of BPD model in SD rats. **(A)** The control group represented rats that had been exposed to indoor air. The experimental rats were exposed to hyperoxia (85% O_2_). The weight of each rat at each time point was recorded. **(B)** Lung histological alterations visualized with H&E staining. From day 7, the number of alveoli and the number of alveolar spaces in the control group increased significantly, while the number of alveoli decreased and the thickness of alveolar walls increased in the BPD model group. **(C)** RAC in the control group was significantly higher than that in the bronchopulmonary dysplasia group (BPD) on days 7, 14, and 21. However, there was no significant change on days 1 and 3. All data are presented as the mean ± SEM of three experiments. *P < 0.05, **P < 0.01, ***P < 0.001.

The mean body weight of SD pups in the normoxic and hyperoxic groups was recorded on days 1, 3, 7, 14, and 21. On Day 1, the weight gain showed no statistical difference between the experimental group and the control group. However, rats in the high-oxygen group began to drop on Day 2, and the difference remained obvious till the last day of survival. The final weight of Day 21 was statistically different between the hyperoxic group and the control group (Figure 8A).

Sections were stained with H&E. Lung morphologic changes were evaluated using light microscopy. Compared with the normoxic group, hyperoxic group demonstrated histological features of decreased septum, enlarged distal cavity and reduced complexity (Figure 8B). To quantify the reduction in the number of alveoli, RAC was performed. There was no significant difference in RAC between the two groups on Days 1 and 3. However, on Days 7, 14, and 21, RAC was significantly larger in the control group than in BPD group. The RAC values of rats exposed to high oxygen environment were lower than those exposed to normoxic environment. Neonatal rats exposed to hyperoxia showed simplified alveolar tissue morphology and decreased RAC, similar to those of children with BPD (Coalson, 2003). These results demonstrate the successful establishment of the animal model of BPD (Figure 8C).

### MRNA and Protein Expression Levels of Inflammatory Cytokines and Oxidative Stress Indicators in Rats

**FIGURE 9:**
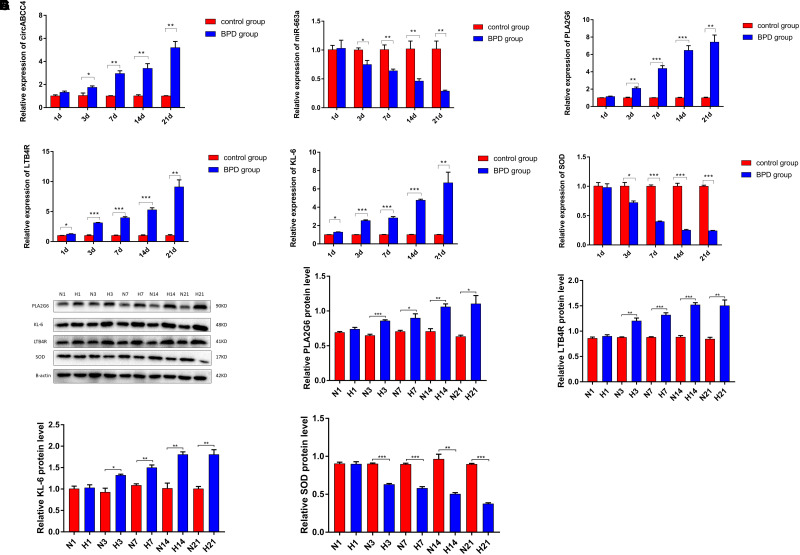
Expression levels of inflammatory factors and oxidative stress indicators in lung tissues were measured. **(A–F)** Relative expression of circABCC4, miR-663a, PLA2G6, LTB4R, KL-6, and SOD mRNA was detected by qRT-PCR in lung tissues. **(G)** Western blotting of PLA2G6, KL-6, LTB4R, and SOD in lung tissues. Protein levels have been quantified. All data are presented as the mean ± SEM of three experiments. *P < 0.05, **P < 0.01, ***P < 0.001.

On Day 1, there was no significant difference in mRNA and protein levels of PLA2G6, LTB4R, KL-6, and SOD between the two groups of lung tissues. From Day 3, the expression levels of all indicators in the control group remained basically unchanged, while the mRNA and protein expressions of PLA2G6, LTB4R, and KL-6 in the BPD group increased gradually, and that of SOD decreased gradually. In addition, this difference between the two groups became more significant as the duration of high oxygen exposure was prolonged (Figures 9A–G).

### Distribution of PLA2G6, LTB4R, KL-6, and SOD

**FIGURE 10:**
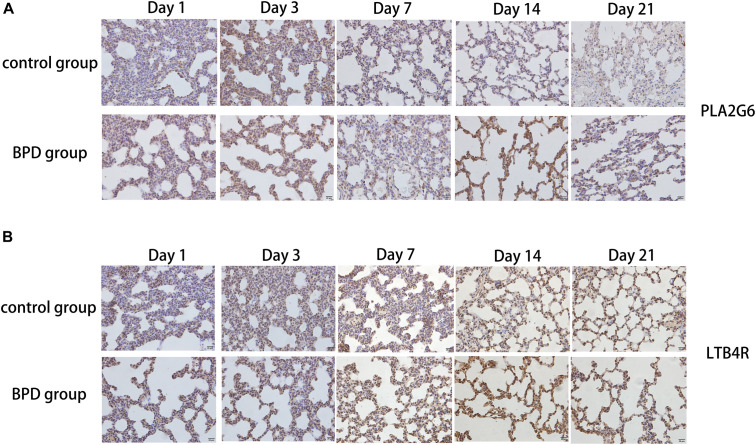
Detection of the expression of PLA2G6 and LTB4R using immunohistochemical staining of rat lungs exposed to room air or hyperoxia for 1, 3, 7, 14, 21 days (magnification, x400). **(A,B)** The results revealed that the expression of PLA2G6 and LTB4R were abundant in the alveolar and bronchiolar epithelial cells of the rats exposed to hyperoxia, while the control rats exhibited weak immunoreactivity for these molecules.

**FIGURE 11:**
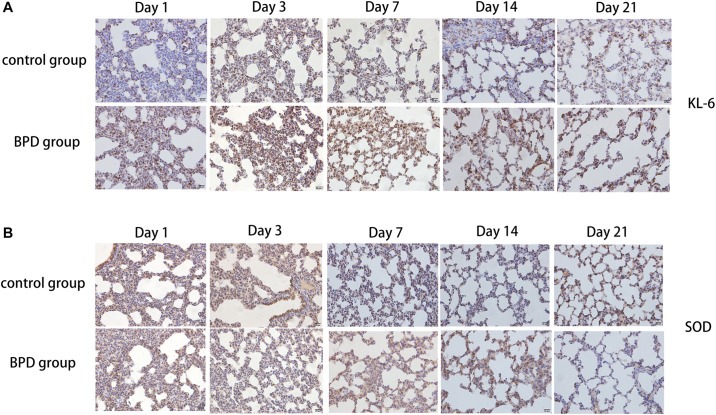
Detection of the expression of KL-6 and SOD using immunohistochemical staining of rat lungs exposed to room air or hyperoxia for 1, 3, 7, 14, 21 days (magnification, x400). **(A)** The results revealed that the expression of KL-6 was abundant in the alveolar and bronchiolar epithelial cells of the rats exposed to hyperoxia, while the control rats exhibited weak immunoreactivity for these molecules. **(B)** The results showed that SOD expression was abundant in alveolar epithelial cells and bronchial epithelial cells, while the immune response to these molecules was weak in rats exposed to high oxygen.

Immunohistochemical results showed that compared with the rats exposed to normal air, alveolar septal arrest in the lung tissue of rats exposed to hyperoxic environment was characterized by increased alveoli and heterogeneous alveoli. PLA2G6, LTB4R, and KL-6 were abundantly expressed in rat tissues exposed to hyperoxia, and SOD expression was reduced (Figures 10A,B). In contrast, the rats in the control group showed weaker immunoreactivity to these molecules. PLA2G6, LTB4R, KL-6, and SOD were preferentially localized to alveolar epithelial cells and bronchial epithelial cells. It is suggested that hyperoxia should increase the content of inflammatory factors in alveoli, and mainly alveolar expansion and simplified epithelial cells (Figures 11A,B).

### Apoptosis Detected by TUNEL Method

**FIGURE 12:**
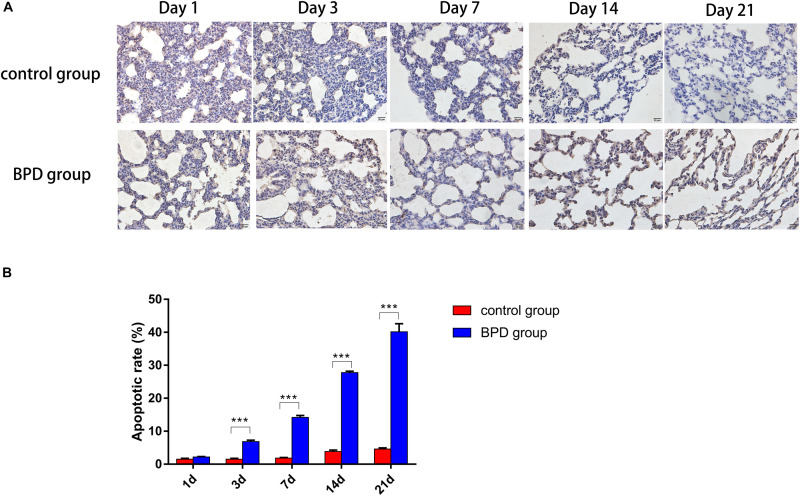
TUNEL staining demonstrated a significant increase in TUNEL-positive cells (magnification, x400). **(A)** Increased numbers of epithelial and endothelial cells were apoptotic in the hyperoxia group, compared with the control group. **(B)** Apoptotic cell indices of neonatal rats were increased gradually over the duration of hyperoxia exposure, and the percentages of apoptotic cells were significantly higher, compared with the control group. All data are presented as the mean ± SEM of three experiments. *P < 0.05, **P < 0.01, ***P < 0.001.

This experiment was performed to determine whether hyperoxia exposure can cause apoptosis in the lungs. After exposure to high oxygen or indoor air for 1, 3, 7, 14, and 21 days, TUNEL analysis was used to detect the state of apoptosis. Basically no TUNEL-positive cells were detected in the control group, but many positive cells were observed in the hyperoxia group. The increase in the number of apoptotic cells was in parallel with the increase in PLA2G6-positive cells, which further indicates that after exposure to hyperoxia, the inflammatory factors-start to mediate the apoptosis of rat lung cells (Figures 12A,B).

## Discussion

Premature babies show high predisposition to BPD, a critical illness lacking effective methods for prevention and treatment (Chen et al., 2017). In 1967, Northway et al. first discovered and reported “classic BPD” secondary to severe NRDS with inflammation as the main manifestation (Northway et al., 1967). With antenatal corticosteroids and before-birth application of exogenous alveolar active substances, the survival rate of very low birth weight infants has been greatly improved. However, instead of being reduced, the overall incidence of BPD has kept rising gradually. Unlike “classic BPD,” the “new BPD” is clinically characterized with lung developmental retardation, moreover, use of glucocorticoids can cause atrophy of the nervous system in children, even cerebral palsy (Day and Ryan, 2017). BPD occurs with a variety of pathogenic factors, but the pathogenesis remains largely unveiled. In the later stage of lung development, secondary septations and distal microvascular maturation maximize the lung surface area for the exchange between the inhaled oxygen and blood. Once this stage is interrupted, the exchange becomes deficient, which leads to BPD in which alveolar surfactants decrease and pulmonary inflammation forms (Kalikkot Thekkeveedu et al., 2017). This study showed that certain circRNAs were upregulated and their sponged miRNAs were downregulated. Modulating these circRNAs, miRNAs or target genes could inhibit the inflammatory response and oxidative stress, thereby reversing the hyperoxia-induced BPD.

CircRNAs and miRNAs are non-coding RNAs involved in the proliferation, differentiation and apoptosis of cells. CircRNAs are a large, conserved, non-coding RNA family that is produced by a non-canonical reverse splicing between covalent bonds between the 5′ and 3′ ends (Li et al., 2018). In recent years, the new research tools, especially high-throughput sequencing and bioinformatics analysis, have confirmed the ubiquity of circRNAs in the development of various diseases (Shen et al., 2019; Zhu et al., 2019). However, their role in BPD is still blurry. In this study, we found a new BPD-related circRNA circABCC4, which was significantly up-regulated in patients’ peripheral blood and the rat model.

It has been suggested that abnormally up-regulated inflammatory factors should disrupt alveolar formation and delay alveolar microvascular maturation. In addition, if the patient is exposed to hyperoxia or mechanical ventilation related infection, BPD may arise (Shahzad et al., 2016). In this study, we used sequencing analysis to obtain expression profiles of the relevant circRNAs in normal neonates and preterm infants receiving mechanical ventilation. The expression of circABCC4 was significantly increased in the peripheral blood of 31 patients with BPD, and positively correlated with the poor long-term prognosis in preterm infants with BPD. Functionally, circABCC4 promotes apoptosis and inhibits cell proliferation both *in vitro* and *in vivo*, suggesting that circABCC4 is a promoter in BPD development. CircABCC4 may be an effective target and prognostic biomarker for the diagnosis and treatment of BPD.

CeRNAs are a type of circRNAs most frequently reported. CeRNAs can regulate the expression of target miRNAs (Piwecka et al., 2017). Many molecular experiments have confirmed that circABCC4 promotes the expression of PLA2G6 in BPD. Rescued experiments have confirmed that PLA2G6 upregulation can reverse the ability of knocked-down circABCC4 to inhibit proliferation and promote apoptosis, and PLA2G6 downregulation can weaken the ability of circABCC4 to enhance proliferation and inhibit apoptosis. In summary, circABCC4 can promote BPD in preterm infants by up-regulating the expression of PLA2G6.

According to the previous experimental results, a complex regulatory network of ceRNAs has been proposed. Here, bioinformatics analysis showed that circABCC4 and PLA2G6 share the MRE of miR-663a, suggestive of the presence of circABCC4/miR-663a/PLA2G6 axis (Supplementary Figure S1C). The RIP experiment confirmed targeting relationship between circABCC4 and miR-663a. Similarly, luciferase reporter assays have shown that miR-663a directly inhibits the expression of PLA2G6 by binding to the 3′-UTR of PLA2G6. For the first time, we found that miR-663a was down-regulated and its downregulation was correlated with the severity of clinicopathological features, suggesting that miR-663a may have protective effects in BPD. Combining these results, we demonstrated that circABCC4, by acting as a miR-663a sponge, increased its target gene PLA2G6 expression, thereby promoting the progression of BPD.

PLA2G6 encodes A2 phospholipase, an enzyme that catalyzes the release of fatty acids from phospholipids. A2 phospholipase is reported to play an important role in the occurrence and development of inflammatory diseases (Grissom et al., 2003; Masuda et al., 2005; Rastogi and McHowat, 2009; Jaburek et al., 2013). In the BPD model, PLA2G6 expression was up-regulated, consequently promoting the expression of inflammatory factors and leukotrienes, accelerating the apoptosis and inhibiting the proliferation of cells (Rastogi and McHowat, 2009). In addition, PLA2G6 catalyzes the cleavage of phospholipid membranes, which are the most important inflammatory mediators of acute lung injury (Shu et al., 2017). The phospholipase A2 isotype is associated with the pathophysiology of inflammatory lung disease. It is produced by alveolar macrophages and other lung cells in the process of inflammatory response, and can promote lung injury by destroying pulmonary surface active substances (Kitsiouli et al., 2015). However, the specific function and mechanism of PLA2G6 in BPD remains unclear. We found that PLA2G6 was up-regulated to promote the apoptosis and inhibit the proliferation of A549 cells in our BPD model. In conclusion, circABCC4 promotes the progression of BPD through the miR-663a/PLA2G6 axis.

Limitations also exist in our study. CircABCC4 can be detected in peripheral blood of patients with BPD, but whether it can be detected in exosomes remains to be confirmed by further experiments. Next, a circRNA may contain many different miRNA binding sites, and a miRNA can also bind multiple circRNAs simultaneously. Therefore, circRNA and miRNA may biologically intersect. In addition, many uncharted circRNAs and their role in the development of BPD remain to be elucidated. Therefore, we need further research to clarify the role of circRNA in BPD.

## Data Availability Statement

The raw data supporting the conclusions of this article will be made available by the authors, without undue reservation.

## Ethics Statement

The animal study was reviewed and approved by the Committee on the Ethics of Animal Experiments of The First Affiliated Hospital with Nanjing Medical University.

## Author Contributions

XC contributed to the conception and design of the study. YC participated in the drafting of the article. YC, DF, JC, and HZ carried out the experiments. ZS, XZ, and BL contributed to all figures and tables. XC, HL, SW, and YY revised the manuscript. AB and DP contributed to data collection and analysis. All authors have read and approved the final manuscript.

## Conflict of Interest

The authors declare that the research was conducted in the absence of any commercial or financial relationships that could be construed as a potential conflict of interest.

## Footnotes

1 http://www.targetscan.org

2 http://www.circbase.org

## Supplementary Material

Supplementary Figure 1 **(A)** ceRNA analysis of hsa circRNA 0030605. hsa circRNA 0030605, 57 miRNAs and 14 target genes are involved in the ceRNA network. Solid lines represent the relationship between two nodes. **(B)** The putative binding site of miR-663a and PLA2G6 and the Mut sequence of PLA2G6 predicted by TargetScan. **(C)** circABCC4 up-regulates PLA2G6 by inhibiting miR-663a to promote the process of BPD.
