# Supplementary figures and images for: Promotion of Bronchopulmonary Dysplasia Progression Using Circular RNA circabcc4 via Facilitating PLA2G6 Expression by Sequestering miR-663a
Source: Front Cell Dev Biol. 2020 Oct 27;8:585541. doi: 10.3389/fcell.2020.585541 (PMC7654334; doi:10.3389/fcell.2020.585541)

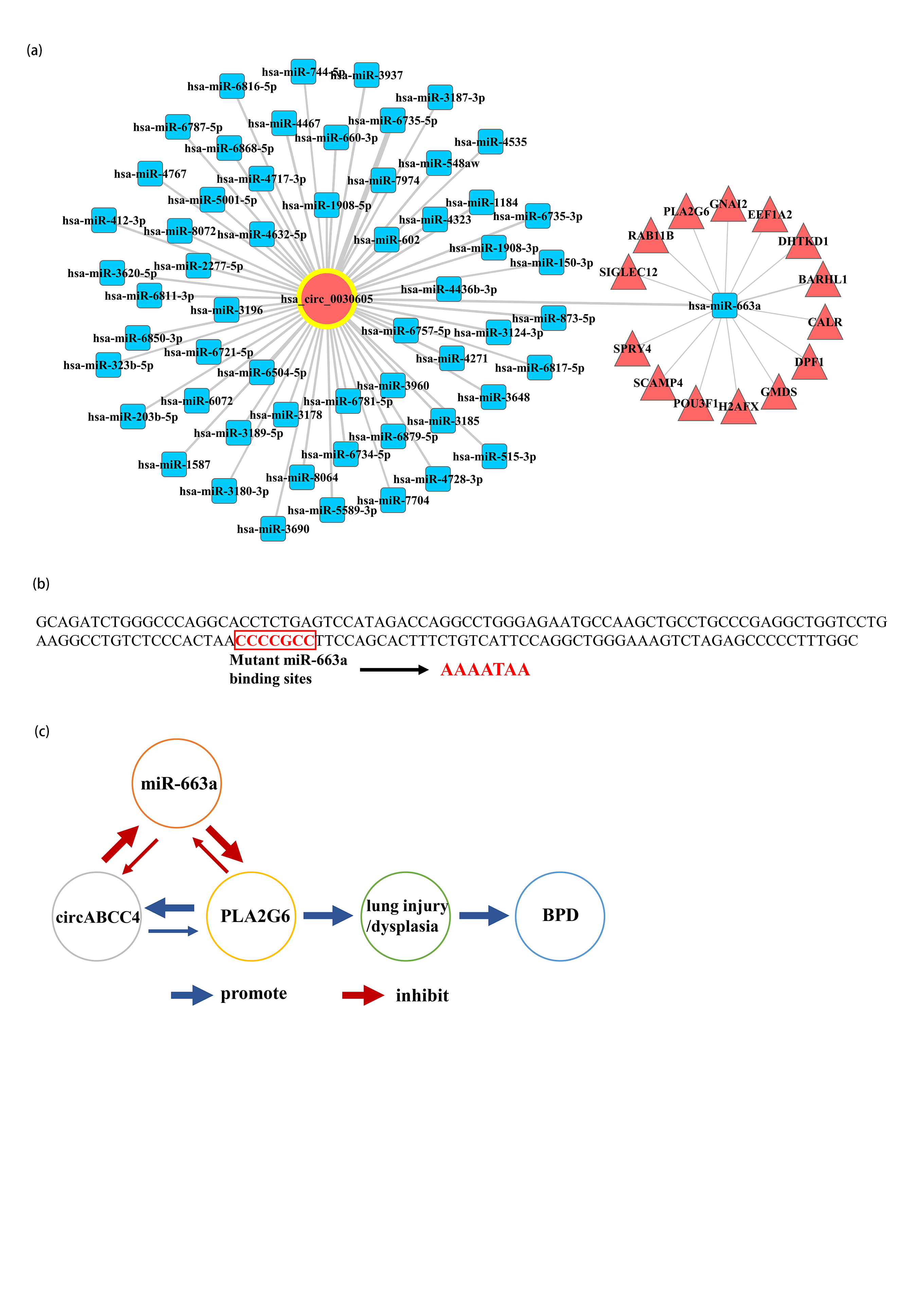

Supplement: Supplementary Figure 1 — (A) ceRNA analysis of hsa circRNA 0030605. hsa circRNA 0030605, 57 miRNAs and 14 target genes are involved in the ceRNA network. Solid lines represent the relationship between two nodes. (B) The putative binding site of miR-663a and PLA2G6 and the Mut sequence of PLA2G6 predicted by TargetScan. (C) circABCC4 up-regulates PLA2G6 by inhibiting miR-663a to promote the process of BPD. [file Figure_1.TIF]
